# Preparation and Characterization of Silica-Enoxil Nanobiocomposites

**DOI:** 10.1186/s11671-016-1287-y

**Published:** 2016-02-04

**Authors:** Pavlo O. Kuzema, Iryna V. Laguta, Oksana N. Stavinskaya, Olga A. Kazakova, Mykola V. Borysenko, Tudor Lupaşcu

**Affiliations:** Chuiko Institute of Surface Chemistry, National Academy of Sciences of Ukraine, General Naumov Street., 17, Kiev, 03164 Ukraine; Institute of Chemistry, Academy of Sciences of Moldova, Academiei Str., 3, 2028 MD Chisinau, Republic of Moldova

**Keywords:** Adsorption, Antioxidant, Biomolecules, Composite, Desorption, Enoxil, Fumed silica, 68.43.Fg, 81.07.Wx, 83.80.Ab

## Abstract

Silica-Enoxil nanobiocomposites with 13 %w of Enoxil were prepared either by mechanical mixing of corresponding powders or by sorptive modification of fumed silica powder with aqueous Enoxil solution under fluidized bed conditions. The interaction of fumed silica with Enoxil and the properties of silica-Enoxil composites have been investigated using IR spectroscopy, thermogravimetric analysis, and quantum chemistry methods, as well as by means of water absorption, Enoxil desorption, and 2,2-diphenyl-1-picrylhydrazyl (DPPH) test. It has been shown that the main biomolecules of Enoxil composition interact with silica involving their hydroxyl groups and surface silanol groups. The water absorption of silica-Enoxil nanocomposites was found to be less than that for the individual components. The Enoxil biomolecules are readily and completely desorbed from silica surface into water, and the antioxidant activity of desorbed Enoxil is practically the same as that for the just dissolved one.

## Background

Recently, much attention of researchers and food manufacturers is focused on a comprehensive study of bioactive compounds of plant origin, in particular, natural polyphenols [1]. The polyphenols represent different types of chemical compounds including catechins, proanthocyanidins, anthocyanins, gallotannins, ellagitannins, flavonol glycosides, hydroxycinnamoyl esters, lignoids, stilbenoids, etc. Plant polyphenols are multifunctional compounds capable to act as the reducing agents, hydrogen-donating antioxidants, and singlet oxygen quenchers. One of the most attractive properties of polyphenols, from the viewpoint of the advantages for the human health, is their ability to exhibit the antioxidant properties. Antioxidants, i.e., the compounds capable of inhibiting or preventing the substrate oxidation [2, 3], are widely used in medicine, cosmetology, and food industry.

Plants, fruits, and vegetables are known to be the sources of polyphenols. Also, grape seeds contain the polyphenol compounds in large quantity. Polyphenol compounds extracted from grape seeds are called oenotannins. Оenotannins are condensed tannins (proanthocyanidins) exhibiting not only strong antioxidant, but also fungicide and antibacterial action. These compounds are widely used in wine-making to improve the wine sensory properties [4]. Oenotannins are composed of flavan-3-ol monomer subunits, such as catechin, epicatechin, and their gallates. Their composition varies from monomers to decamers of catechin or epicatechin. They dissolve in ethanol, methanol, ethylacetate, acetone, and other organic solvents. Insolubility of the majority of these compounds in water restrains their wide use in various branches of industry. For increasing the solubility in water, оenotannins were chemically treated with H_2_O_2_, which has led to depolymerisation of catechin and epicatechin oligomers, and as a result, novel hydrophilic product (Enoxil) has been obtained [5].

According to [6], the Enoxil is a mixture of monomeric derivatives of catechin and epicatechin in the free form and esterified with gallic acid as well as with peroxidic compounds. It is a preparation with high antioxidant activity and amplified therapeutic properties [7, 8]. Most of Enoxil’s compounds have three or more active hydroxyl groups providing them with both high antioxidant and hydrophilic properties. Due to high content of hydrophilic groups, Enoxil preparation is also characterized by enhanced hygroscopicity, which, in turn, requires special conditions for its storage or inclusion in formulation preventing excess water uptake. Probably, such formulation may be designed as silica-Enoxil composite, with silica playing a role of a carrier of bioactive molecules. According to [9, 10], silica has been widely used to integrate in one form several active substances with different mechanisms of action to increase their bioavailability and stability and to provide prolonged release of active substances. One can suggest that keeping the Enoxil preparation in the form of silica-Enoxil composites may improve Enoxil storage stability due to inclusion of Enoxil hydroxyl groups in interaction with silica silanol groups.

The aim of this work was to prepare the silica-Enoxil nanobiocomposites, to study the silica-Enoxil interaction, and to examine the effect of silica on Enoxil water absorption and antioxidant properties.

## Methods

Enoxil powder (produced at the Institute of Chemistry, Academy of Sciences of Moldova) was either mechanically mixed with fumed silica A300 (*S*_sp_ 300 m^2^/g, Kalush, Ukraine) or immobilized on silica via its modification with aqueous solution of Enoxil in a reactor with a mixer, followed by water removal. The Enoxil-to-silica ratio was 1:6.7 (i.e., 13 %w of Enoxil) which, in the case of planar arrangement of biomolecules on silica surface, provides approximate theoretically calculated monolayer.

IR spectra were registered using a Specord M80 spectrometer (Germany) within the interval of wavenumbers 4000–1200 cm^−1^. For the spectra recording, all the samples were compacted into thin rectangular plates of 8 × 28 mm size and of 15 ± 0.5 mg mass, the pure Enoxil sample being preliminary mixed with KBr in a ratio of 1:9.

Thermogravimetric (TG)/differential thermogravimetric (DTG) analysis of the samples was performed using a MOM Q-1500 derivatograph (Paulik and Erdey, Hungary) in a temperature interval of 20–1000 °С. The analysis was run in air atmosphere, on samples (about 40–200 mg) placed in alumina crucibles, at a heating rate of 10 °С min^−1^. The physical water content was determined by dividing the area of the split peak centered at 100 °С into the total area of peaks obtained by Gauss multipeak fitting of DTG curve.

The quantum chemical methods were used for the study of silica-Enoxil interaction. Full optimization of catechin, epicatechin, gallic acid, and model silica clusters consisting of 36 SiO_4/2_ tetrahedrons was calculated using the semiempirical РМ3 method (GAMESS (current versions)) [11]. The solvation model SM5.42 and РМ3 method (GAMESOL program package, Version 3.1 [12]) were used to study the solvent effects for molecules, clusters, and their complexes.

The capability of Enoxil and silica-Enoxil composites to absorb water from the gas phase was determined gravimetrically by the sample mass change before and after holding in an exsiccator. The samples of certain mass were placed into the weighing bottles, which then were placed into a water exsiccator and held there at room temperature for 1–2200 h. For the determined water absorption parameter (Δ*m*/*m*), standard error of the mean was less than 3 % of its value; scattering of the data corresponding to 95 % confidence interval did not exceed 13 % of the mean.

Enoxil desorption into water was studied under the conditions of constant solution volume at an adsorbent-to-solution ratio of 0.1 g/l. Enoxil concentration in aqueous solution was determined spectrophotometrically. The relative error of desorption value determination was less than 3 %.

Stable radical 2,2-diphenyl-1-picrylhydrazyl (DPPH) (Merck, Germany) was used to evaluate the sample’s antioxidant activity [13]. One milliliter of the solution investigated was added to 4 ml of 0.08 mM solution of DPPH. The concentration of stable radicals at various times after the start of the reaction was determined spectrophotometrically by the change in optical density at the absorption maximum of 520 nm. As control, the solution with the same concentration of DPPH, but without the antioxidant, was used.

UV/Vis spectra were recorded on a Perkin Elmer Lambda 35 UV/Vis double-beam Spectrophotometer at 25 °C in the wavelength range of 200–800 nm using a quartz cuvette (pathlength of cuvette was 10 mm). The accuracy of the spectrophotometric measurements was within 1 %.

## Results and Discussion

**Fig. 1:**
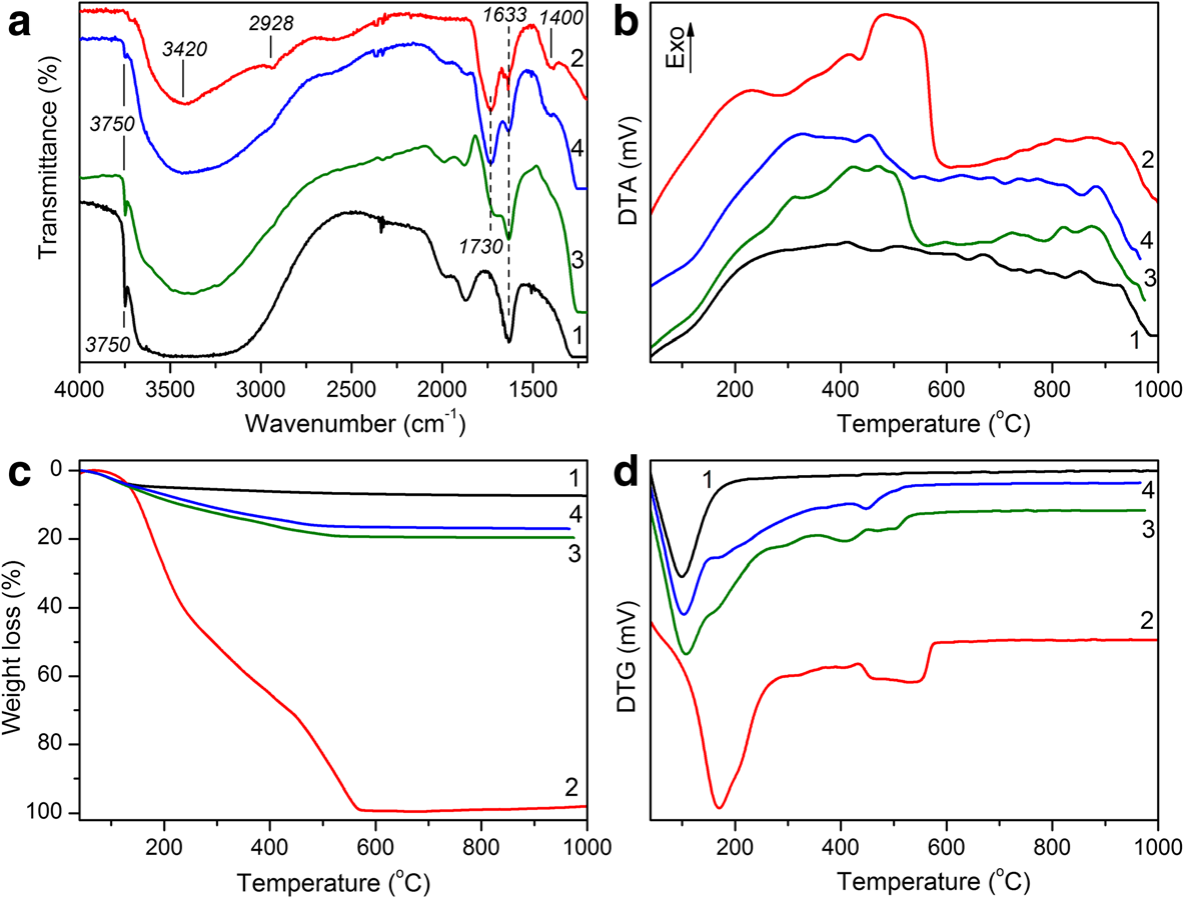
IR spectra (**a**), DTA (**b**), TG (**c**), and DTG (**d**) curves. *1*—pristine silica A300; *2*—pure Enoxil; *3*—silica-Enoxil composite prepared by mechanical mixing; *4*—silica-Enoxil composite prepared by Enoxil immobilization on silica surface

**Fig. 2:**
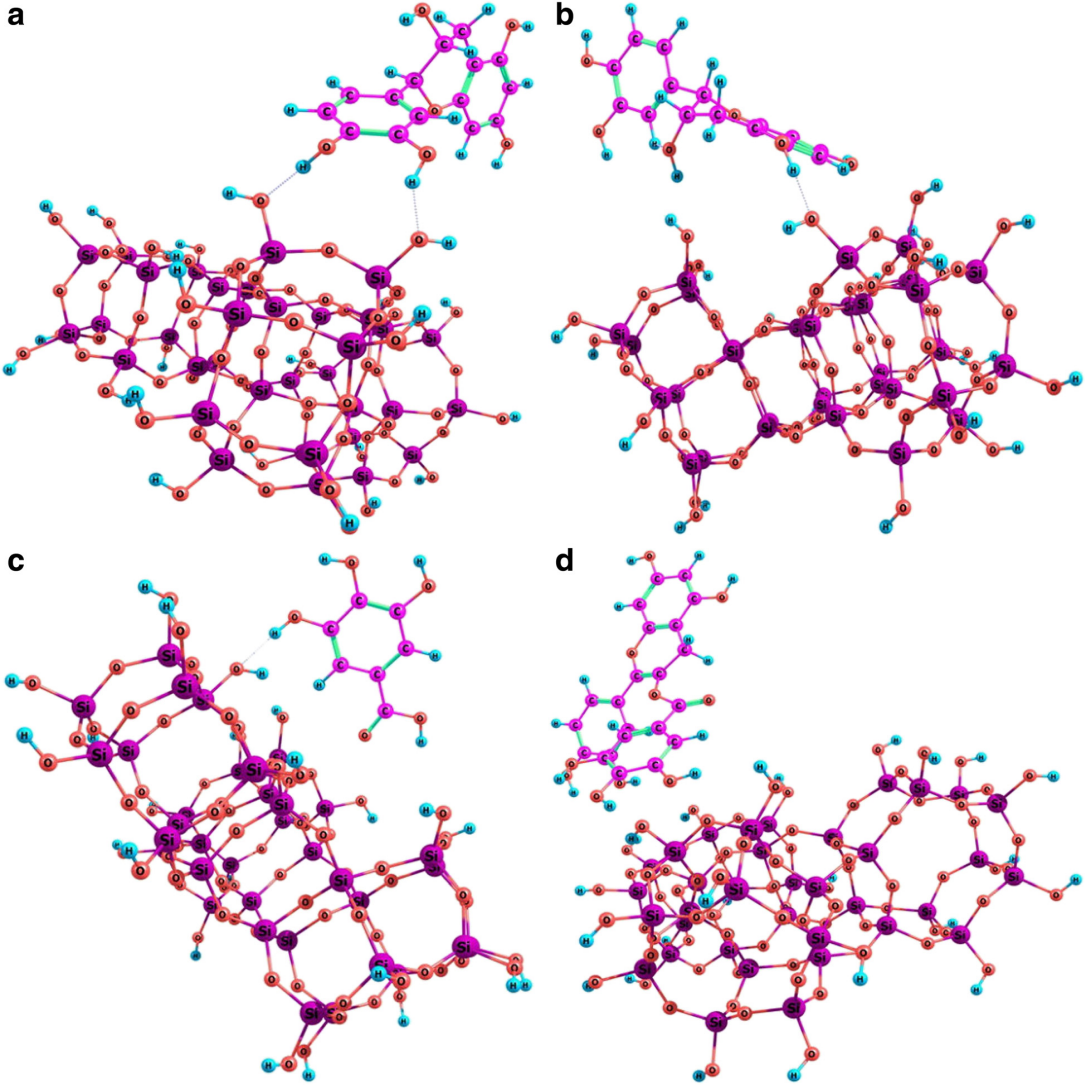
Model of adsorption complex of biomolecules on silica surface. **a** (+)-catechin; **b** (−)-epicatechin; **c** gallic acid; and **d** (+)-catechin gallate

IR spectra of fumed silica A300, Enoxil, and silica-Enoxil nanocomposites obtained by mechanical mixing (Composite 1) and Enoxil immobilization (Composite 2) are given in Fig. 1a. The spectrum of pristine silica (curve 1) shows the narrow absorption band at 3750 cm^−1^, the broad band at 2560–3672 cm^−1^, and the distinct band at 1633 cm^−1^ assigned to O–H stretching vibrations in free (isolated) silanol groups, O–H stretching vibrations of water molecules adsorbed at silica surface, and O–H deformation vibrations in water molecules, respectively [14]. The Enoxil spectrum (curve 2) shows the broad band with a maximum at 3420 cm^−1^ due to O–H vibrations and strong hydrogen bindings, the band at 2928 cm^−1^ related to the symmetric –C–H– stretching vibrations of CH_2_ and CH_3_ groups, the band at 1730 cm^–1^ corresponding to C=O stretching vibrations and associated with the presence of carboxylic acids (dimmers), the band at 1630 cm^−1^ assigned to the symmetric –C–O– stretching vibrations in the aromatic rings, and the band at 1400 cm^−1^ attributed to the deformation vibrations of the C–C bonds in the phenolic groups [6, 15]. By analyzing of the IR spectra of silica-Enoxil nanocomposites (curves 3–4), one can see that the band with a maximum at 3750 cm^−1^ in the spectra of both composite samples is reduced in comparison with that for pristine silica (curve 1). The results suggest that free surface silanol groups are involved in the interaction with biomolecules even in the case of a mechanical mixture; however, no monolayer was formed even when silica was modified with Enoxil solution. The latter can be explained by non-planar arrangement of biomolecules on the silica surface. The carboxylic as well as carbonyl groups of biomolecules are not involved in the formation of adsorption complexes with the silica surface; the band at 1730 cm^−1^ in IR spectra of silica-Enoxil samples remains undisturbed as compared to the one for pure Enoxil spectrum. These two statements above are confirmed by the results of quantum chemical calculations of optimized geometry for adsorption complexes of the most representative Enoxil biomolecules ((+)-catechin, (−)-epicatechin, gallic acid, and (+)-catechin gallate) with silica surface (Fig. 2). In all cases, a non-planar orientation of adsorbate molecules toward the silica surface is the most favorable, and carbonyl as well as carboxyl groups of biomolecules are most likely not involved in the formation of adsorption complexes.

Thermogravimetric analysis (TGA) of pristine silica A300 (Fig. 1b–d, curve 1) shows one distinct weight loss peak centered at 100 °C and related mostly to evaporation of physically adsorbed water. This process is accompanied by a slight endo-effect. Further, slight weight loss is observed which is associated with removal of water formed as a result of surface silanols condensation. The total weight loss was 7.4 %, the physical water content being about 5.9 %.

TGA of Enoxil (Fig. 1b–d, curve 2) shows several distinct thermal zones: the first one (ca. 48 %w, 100–300 °C with the weight loss peak centered at 170 °C) is due to formation of volatile organic species, water, and carbon dioxide as a result of depolymerisation, dehydroxylation, and decarboxylation processes [15, 16]; the second one (ca. 20 %, 300–440 °C) is probably related to further dehydroxylation [16]; and the third one (ca. 29 %, 440–580 °C) is associated with H_2_O, CO, and CO_2_ loss as a result of thermal oxidation of residual carbon-containing species [17]. All the three stages of the weight loss are accompanied with exo-effect, the one for the latest stage being the most pronounced. The total weight loss is 100 %, already at 580 °C.

Thermogravimetric analysis demonstrates a discrepancy in thermal behavior of silica-Enoxil Composites 1 and 2 prepared in two different ways (Fig. 1b–d, curves 3–4). In the case of Composite 1, the weight loss peaks as a result of Enoxil degradation are more pronounced than in the case of Composite 2, and one may state that DTG and differential thermogravimetric analysis (DTA) curves of the former are somewhat a sum of those for pure Enoxil and pristine silica. This is not the case for Composite 2, where there is a pronounced influence of the silica surface on the proceeding of Enoxil thermal degradation. This influence is reflected in the blurring and shifting of degradation peaks, and the total weight loss (17.0 %) is less than the theoretical one in the case of 13 %w Enoxil-containing silica with independently degrading components (19.4 %) calculated from the TG curves for pristine silica and for pure Enoxil. For Composite 1, this value is close to theoretical one (19.7 %).

Free energy of interaction (Δ*G*) of (+)-catechin and (−)-epicatechin, gallic acid, and (+)**-**catechin gallate with the model silica clusters (Fig. 2), calculated using the quantum chemistry methods, was −44.2, −37.4, −31.5, and −54.4 kJ/mol, respectively. By comparing these values with those for the energy of local interactions between the individual O–H groups of the molecules under study and O–H groups of silica (Table 1), one may conclude that on the average, two O–H groups of the biomolecules are involved in the formation of adsorption complexes on the silica surface. This conclusion is in agreement with the distance between involved O–H groups of the biomolecules (0.9–1.2 nm) and the most probable distance between free O–H groups of the silica surface (0.7 nm). The rest of the hydroxyl groups of the biomolecules were found not to participate in adsorption interactions; optimization of the adsorption complexes’ geometry in any case did not result in planar arrangement of the biomolecules on the surface.

**Table 1:** Free energy of interaction (kJ/mol) between biomolecules functional groups and silica in aqueous medium

Group	(+)-catechin	(−)-epicatechin	Gallic acid
3-OH	−7.1	−16.7	−0.2
4-OH			−11.1
5-OH	−4.2	−13.1	−15.2
7-OH	−0.9	−7.6	
3'-OH	−10.4	−8.6	
4'-OH	−13.3	−13.8	
COOH			−7.2

**Fig. 3:**
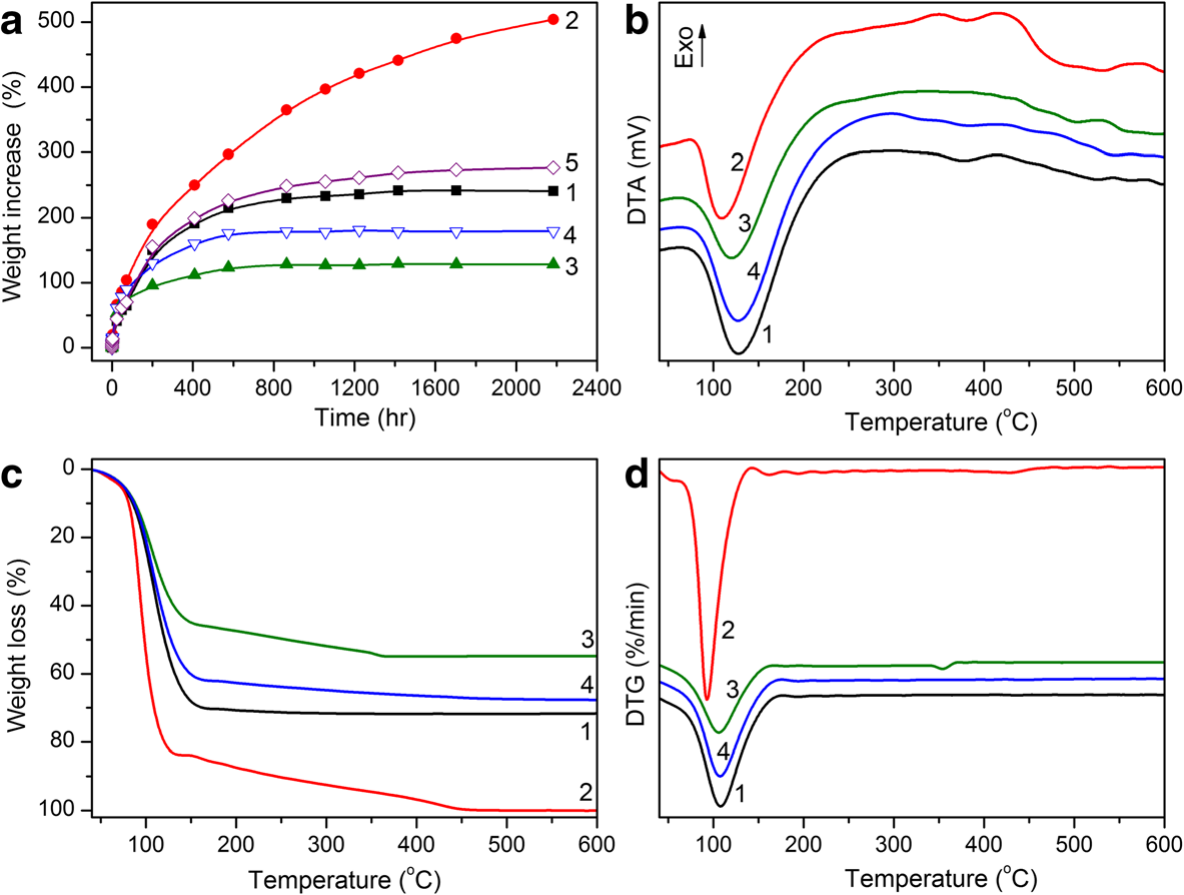
Mass increase (**a**), DTA (**b**), TG (**c**), and DTG (**d**) curves for the samples after water absorption. *1*—pristine silica A300; *2*—pure Enoxil; *3*—silica-Enoxil composite prepared by mechanical mixing; *4*—silica-Enoxil composite prepared by Enoxil immobilization on silica surface; *5*—theoretical curve calculated from curves 1 and 2 for 87 %w of silica and 13 %w of Enoxil

The results of the quantum chemical calculations (Fig. 2) indicate that it is the hydroxyl groups of adsorbate biomolecules and silica surface that participate in the formation of adsorption complexes. To prove this finding experimentally as well as to elucidate the composites’ storage stability under air moisture conditions, we studied the water absorption by the nanocomposites as well as the individual components. The data on water absorption from the gas phase by the pristine silica, pure Enoxil, and silica-Enoxil nanocomposites are given in Fig. 3a (curves 1–4). For the comparison, the theoretical curve describes how much water would be absorbed by the sample containing 87 %w of silica and 13 %w of Enoxil (i.e., the silica-to-Enoxil ratio equivalent to that for the silica-Enoxil composites) in the case when individual components do not affect the water absorption by each other. During the first hour, the water absorption by the composites was higher than that for the pristine silica and pure Enoxil. Then, it slowed down and after several days became less than that in the case of the individual components. The amount of water absorbed by the Enoxil still continued to grow even after 3 months of holding in an exsiccator, whereas the appropriate curves for the silica and silica-Enoxil composites became saturated after 30 days. Apparently, the Enoxil dispersion along the silica surface allows higher access of O–H groups in its biomolecules for interaction with water than in the case of the Enoxil powder where the biomolecules are bound in agglomerates/particles, so that the majority of hydroxyl groups are not capable to interact with water. In the course of time, the Enoxil agglomerates/particles are dissolved in the absorbed water releasing new hydroxyl groups for interaction with water, while some portion of O–H groups of immobilized biomolecules are involved into interaction with silica, so that the total amount of free hydroxyls in the composite is less than in the sum of its individual components. After 3 months of holding in an exsiccator with saturated water vapors, the composites kept their powdered form while the pure Enoxil sample turned into the solution.

The samples being held for 3 months in a water exsiccator were then studied using thermogravimetric analysis. As Fig.3b–d shows, the main region of weight loss (up to 150 °C) corresponds to evaporation of water, accompanied by the strong endo-effect visible on DTA curves. The data on water absorption are summarized in Table 2 and demonstrate good agreement between the amount of water absorbed by the samples, determined using the gravimetric and thermogravimetric analysis. While the less amount of the absorbed water by the composites than by the individual components could be explained by an exclusion of some part of the hydroxyl groups involved in the silica-Enoxil binding, it was surprising to find out that the composite obtained by mechanical mixing of silica and Enoxil powders absorbed a less amount of water than the one obtained by silica modification with Enoxil aqueous solution and subsequent elimination of the solvent. This is probably due to a decrease in the interaggregate porosity in the case of mechanical mixture—mechanical mixing could change the morphology of branched fumed silica agglomerates.

**Table 2:** The results of water absorption studies by gravimetric and thermogravimetric analysis

Sample	Mass (*m*), mg	Total weight loss, % (by TG)	Physical water content, % (by DTG)	Water absorption, % (Δ*m*/*m* by DTG)	Water absorption, % (∆*m*/*m* by GA)
A300	187.5	7.4	5.9 ± 0.2		
Enoxil	43.3	100	3.1 ± 0.1		
Composite 1	201.8	19.7	5.4 ± 0.1		
Composite 2	202.4	17.0	4.7 ± 0.1		
A300 + water	100	71.0	69.5 ± 1.3	227 ± 8	235 ± 13
Enoxil + water	51.9	100	83.7 ± 1.3	513 ± 16	504 ± 21
Composite 1 + water	92.6	59.1	54.4 ± 1.1	119 ± 5	129 ± 10
Composite 2 + water	101.0	67.8	63.1 ± 1.1	170 ± 6	181 ± 11

**Fig. 4:**
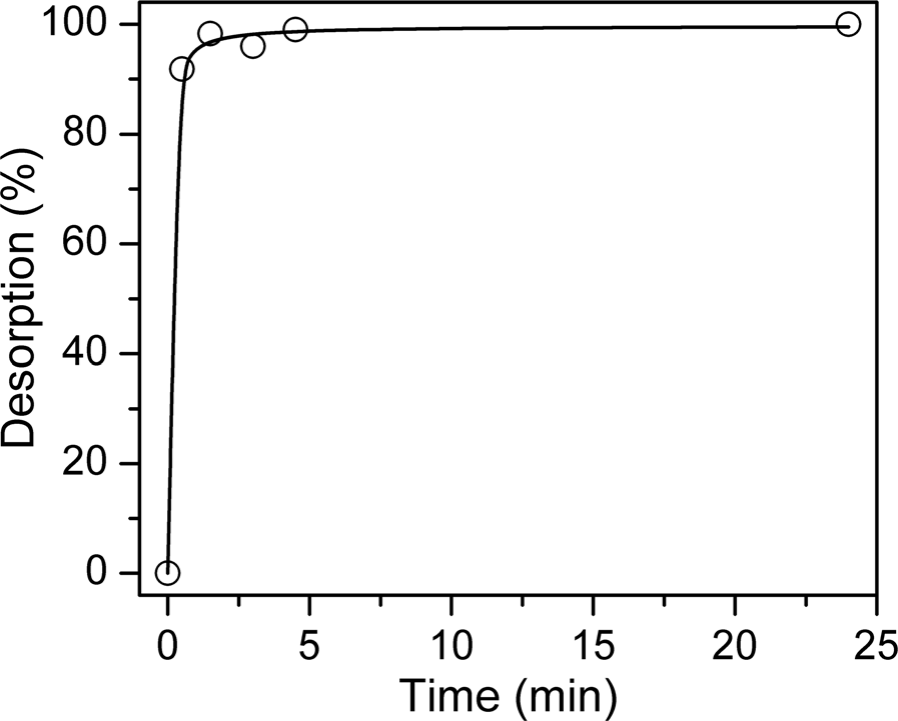
Desorption of immobilized Enoxil into water

**Fig. 5:**
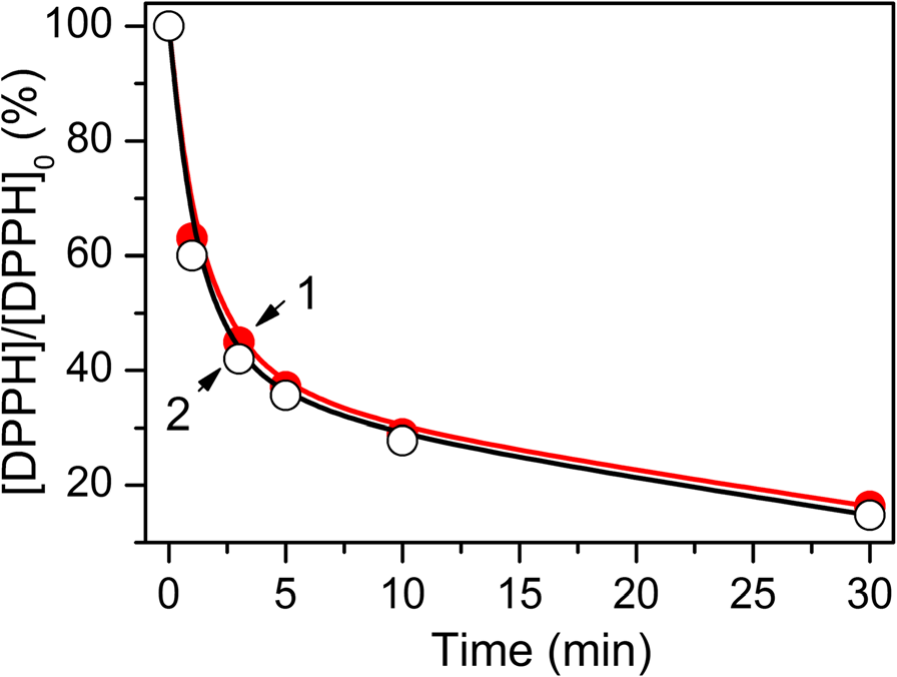
Antioxidant activity by the DPPH assay. *1*—solution of pure Enoxil; *2*—solution of Enoxil desorbed from the silica surface

The data on the Enoxil desorption from the silica surface of Composite 2 into the aqueous medium are given in Fig. 4. As it can be seen, already within 30 min, almost complete desorption of biomolecules from the surface is observed. Upon this, the total amount of the desorbed preparation is in good agreement with the theoretical amount of Enoxil in the composite. The data on the interaction of the stable DPPH radical with the desorbed Enoxil and with the fresh-made Enoxil solution of the equivalent concentration are shown in Fig. 5. It turned out that the antioxidant properties of Enoxil did not change during the process of the composite preparation and during its storage for several months.

## Conclusions

Silica-Enoxil nanocomposites were prepared either by mechanical mixing of Enoxil and fumed silica powders or by Enoxil immobilization on the fumed silica surface from aqueous solution. By means of IR spectroscopy and quantum chemistry studies, it has been found that the main Enoxil compounds interact with the silica surface via their hydroxyl groups; on the average, two O–H groups of biomolecules are involved in interaction with silanol groups. Thermogravimetric analysis of the composites reveals stronger influence of silica on Enoxil thermal degradation in the case of immobilized biomolecules, indicating the higher dispersion of Enoxil along the silica surface than in the case of the silica-Enoxil mechanical mixture. The water absorption of silica-Enoxil nanocomposites was found to be less than that for the individual components. This is an additional evidence that silica-Enoxil interaction involves the hydroxyl groups of each component, which become unavailable for interaction with water. Immobilized Enoxil biomolecules are weakly bound to the silica surface and are readily and completely desorbed into water after 30 min. Enoxil does not lose its antioxidant activity even after being stored in the form of silica-Enoxil composites for several months. Both methods for the preparation of nanobiocomposites are suitable for decreasing Enoxil hygroscopicity and for improvement of its storage stability.

## Abbreviations

%w: weight percent
A300: fumed silica with a specific surface 300 m^2^/g
Composite 1: silica-Enoxil composite prepared by mechanical mixing of corresponding fumed silica and Enoxil powders
Composite 2: silica-Enoxil composite prepared by fumed silica modification with aqueous Enoxil solution under fluidized bed conditions with subsequent water removal
DPPH: 2,2-diphenyl-1-picrylhydrazyl
DTA: differential thermal analysis
DTG: differential thermogravimetric
GA: gravimetric analysis
IR: infrared
*M*: mass (weight)
*S*_sp_: specific surface area
TG: thermogravimetric
TGA: thermogravimetric analysis
UV/Vis: ultraviolet/visible
Δ*G*: the free energy of interaction
Δ*m*: mass (weight) change
